# Green Solvent-Based Dispersive Liquid–Liquid Microextraction Method Coupled with High-Performance Liquid Chromatography for the Determination of Triazole Fungicides in Cereal Samples

**DOI:** 10.3390/foods15173002

**Published:** 2026-08-26

**Authors:** Min Li, Yulin Wang, Huajuan Yin, Xu Jing, Yunlong Li

**Affiliations:** 1 Shanxi Institute for Functional Food, Shanxi Agricultural University, Taiyuan 030031, China; li407267281@163.com; 2 Houji Laboratory in Shanxi Province, College of Food Science and Engineering, Shanxi Agricultural University, Taiyuan 030031, China; 15161869838@163.com (Y.W.); 16635424019@163.com (H.Y.)

**Keywords:** dispersive liquid–liquid microextraction, pesticides, magnetic deep eutectic solvents, liquid-phase microextraction, dispersion

## Abstract

Triazole fungicides (TFs) are widely used in cereal production due to their potent fungicidal activity and broad-spectrum efficacy. Nonetheless, residues of TFs in food products may pose risks to food safety and human health. Therefore, the development of efficient and environmentally friendly sample preparation methods is paramount for the reliable determination of TFs. Herein, a novel green solvent-based dispersive liquid–liquid microextraction method coupled with high-performance liquid chromatography (DLLME-HPLC) was developed for the determination of TFs in cereal samples. The prepared magnetic deep eutectic solvents (MDESs), composed of nonanoic acid and ferric hydroxide, served as green, magnetically responsive extraction solvents, enabling rapid magnetic separation without centrifugation. Four bio-based solvents (BBSs) were investigated as green dispersive solvents to facilitate the dispersion of MDESs and replace conventional toxic organic dispersants, thereby further enhancing the environmental sustainability of the extraction procedure. Owing to the combined effects of hydrophobic interactions, hydrogen-bonding networks, and magnetic responsiveness, the proposed method achieved efficient extraction and rapid phase separation while minimizing solvent consumption and operational complexity. The greenness of the method was evaluated using multiple green analytical chemistry metrics, confirming its low environmental impact, reduced waste generation, and improved operational safety compared with conventional DLLME procedures. Under optimized conditions, the method was successfully applied to determine TFs in rice, wheat, corn, buckwheat, and oat samples, achieving recoveries ranging from 75.0% to 101.9% and relative standard deviations of 1.6–4.8%. The developed DLLME method provides a rapid, sensitive, and environmentally friendly strategy for cereal pesticide residue analysis and expands the application of MDESs and BBSs in green sample preparation.

## 1. Introduction

Triazole fungicides (TFs) are among the most extensively used pesticides for controlling fungal diseases in cereal crops due to their broad-spectrum activity and high efficacy. Notably, their antifungal mechanism primarily involves inhibiting ergosterol biosynthesis, thereby disrupting fungal cell membrane integrity and function [1]. Nevertheless, the widespread, long-term use of TFs inevitably leads to their accumulation in agricultural products. Residual TFs in food products may pose potential risks to human health due to their endocrine-disrupting effects, reproductive toxicity, and potential carcinogenicity. Therefore, the development of sensitive, reliable, and efficient analytical techniques for detecting TFs in cereals is of great importance for ensuring food safety.

The determination of pesticide residues in cereals remains challenging because grain extracts are complex matrices containing large amounts of proteins, lipids, carbohydrates, and other endogenous compounds, whereas pesticide residues are generally present at trace levels [2]. Therefore, sample preparation plays a crucial role in improving analytical sensitivity, selectivity, and accuracy [3,4]. Conventional pretreatment techniques mainly rely on liquid–liquid extraction (LLE) and solid-phase extraction (SPE). However, LLE is labor-intensive and requires significant volumes of organic solvents, often involving additional concentration steps. SPE reduces solvent consumption but often relies on disposable cartridges, leading to higher operational costs and increased waste generation [5]. Conversely, liquid-phase microextraction (LPME) has emerged as an attractive alternative owing to its minimal solvent consumption, operational simplicity, and high enrichment capability [6]. Among various LPME techniques, dispersive liquid–liquid microextraction (DLLME) is one of the most widely adopted approaches due to its rapid mass-transfer kinetics and excellent extraction efficiency [7].

However, the practical application of DLLME remains limited by the challenge of efficiently recovering the finely dispersed extraction droplets. In DLLME, the extractant is dispersed into numerous microdroplets to maximize interfacial contact and enhance mass transfer. Although this strategy significantly improves extraction efficiency, it also complicates subsequent phase separation. Typically, centrifugation is required to facilitate phase separation, which increases analysis time and introduces additional reliance on specialized equipment. To overcome this limitation, magnetic deep eutectic solvents (MDESs), a representative class of magnetic functional solvents, have recently been developed [8]. By incorporating magnetic components into DES systems, MDESs integrate extraction capability with magnetic responsiveness, enabling rapid phase separation under an external magnetic field without the need for centrifugation [9].

However, most reported MDESs are based on ferric chloride-containing systems, which often exhibit relatively weak magnetic responsiveness and inefficient magnetic collection [10,11,12]. Therefore, the development of novel MDESs with enhanced magnetic properties remains highly desirable. In the present study, a novel MDES was constructed using medium-chain fatty acids as hydrogen-bond donors and metal hydroxides as both hydrogen-bond acceptors and magnetic-response sources. Compared with conventional ferric chloride-based MDESs, using iron hydroxides not only enhances magnetic responsiveness and accelerates magnetic separation but also provides additional coordination sites that contribute to the structural stability of the DES. The selected medium-chain fatty acids exhibit suitable hydrophobicity and flexible alkyl chains, facilitating the efficient partitioning of moderately hydrophobic TFs from aqueous media [13]. Furthermore, the synergistic interactions between fatty acids and metal hydroxides establish a stable hydrogen-bonding network, resulting in a functional MDES that simultaneously serves as both an extraction solvent and a magnetic separation medium [14]. This integrated design eliminates the need for toxic chlorinated extractants and post-extraction centrifugation, thereby simplifying the analytical procedure and enhancing its environmental sustainability.

In conventional DLLME, dispersive solvents are commonly used to facilitate the dispersion of extractants into aqueous samples. However, these solvents often exhibit varying degrees of toxicity and environmental concerns. For example, methanol is neurotoxic, acetonitrile may generate cyanide metabolites, and acetone is highly flammable and can cause irritation. The extensive use of these solvents compromises the environmental sustainability of DLLME. Bio-based solvents (BBSs) derived from renewable resources have recently emerged as promising alternatives owing to their low toxicity, biodegradability, and sustainability. Their incorporation into DLLME systems can effectively reduce the environmental and health impacts. BBSs generally exhibit low toxicity, high biodegradability, and favorable environmental compatibility. Representative BBSs, including dimethyl isosorbide (DMI), ethyl lactate (EL), 2-methyltetrahydrofuran (2-MeTHF), and dihydrolevoglucosenone (Cyrene), have attracted increasing attention as environmentally friendly substitutes for petroleum-derived solvents. Their favorable miscibility with both hydrophilic and hydrophobic phases enables efficient dispersion of extraction solvents while minimizing the extraction process’s environmental footprint. Nevertheless, the application of BBSs as dispersive solvents in DLLME remains relatively limited. Therefore, four representative BBSs were investigated in this study as potential green dispersive solvents to further enhance the greenness of the sample preparation procedure. The combination of MDESs and BBSs establishes a fully green DLLME platform with reduced environmental impact, improved operational safety, and enhanced extraction performance.

Here, a green solvent-based DLLME method was developed for the determination of three representative TFs in cereal samples. The effects of various extraction conditions were systematically optimized. The proposed method was subsequently validated using rice, wheat, corn, buckwheat, and oat samples for linearity, sensitivity, accuracy, and precision. Collectively, our findings provide a rapid, sensitive, and environmentally friendly analytical strategy for monitoring triazole fungicide residues in cereal products and further expand the application of MDESs and BBSs in green sample preparation.

## 2. Materials and Methods

### 2.1. Reagents and Materials

Triadimenol (96%), triadimefon (99%), flusilazole (98%), pentanoic acid, hexanoic acid, heptanoic acid, octanoic acid, nonanoic acid, decanoic acid, DMI, Cyrene, 2-MeTHF, EL, methanol, acetonitrile, acetone, ethanol, sodium chloride (NaCl), iron hydroxide (Fe(OH)_3_), nickel hydroxide (Ni(OH)_2_), and cobalt hydroxide (Co(OH)_3_) were purchased from Aladdin Biotechnology Co., Ltd. (Shanghai, China). Corn, wheat, rice, buckwheat, and oat samples were purchased from a local market in Taiyuan, China.

The stock standard solution of TFs (1000 μg mL^−1^) was prepared in acetonitrile and stored at −10 °C in the dark. Working standard solutions at different concentrations were freshly prepared by serial dilution of the stock standard solution with acetonitrile. Briefly, 50 μL of working standard solutions was spiked into 1 g of blank rice, wheat, corn, buckwheat, and oat samples to prepare spiked cereal samples at concentrations ranging from 0.001 to 0.1 μg g^−1^.

### 2.2. Instrumentation

Chromatographic analysis was conducted using an Agilent 1260 Infinity III high-performance liquid chromatography system equipped with a diode array detector (Waldbronn, Germany) and an Agilent HC-C18 column (250 × 4.6 mm, 3 μm). The injection volume was set at 5 μL. Acetonitrile and water (51:49, *v*/*v*) were used as the mobile phase at a flow rate of 1 mL min^−1^, and the column temperature was maintained at 30 °C. The detection wavelength for TFs was set at 220 nm. The representative chromatogram is shown in Figure S1.

### 2.3. Preparation of MDES

The metal hydroxide and fatty acid were mixed at a molar ratio of 5:1 in a conical flask, and the resulting mixture was heated with continuous magnetic stirring in a water bath maintained at 80 °C for 30 min. After completion of the reaction, the MDES was obtained. The viscosity was 8.90 mPa s. Vibrating sample magnetometer analysis is shown in Figure S2.

### 2.4. Sample Preparation Procedure

All experiments were conducted on 1 g of blank cereal samples spiked with 50 µL of mixed standard solution of TFs. One gram of ground cereal sample was accurately weighed into a 10 mL centrifuge tube, followed by the addition of 1500 μL of DMI. After vortex mixing for 1 min, the resulting sample extract was collected and stored for subsequent analysis.

### 2.5. Microextraction Procedure

**Figure 1:**
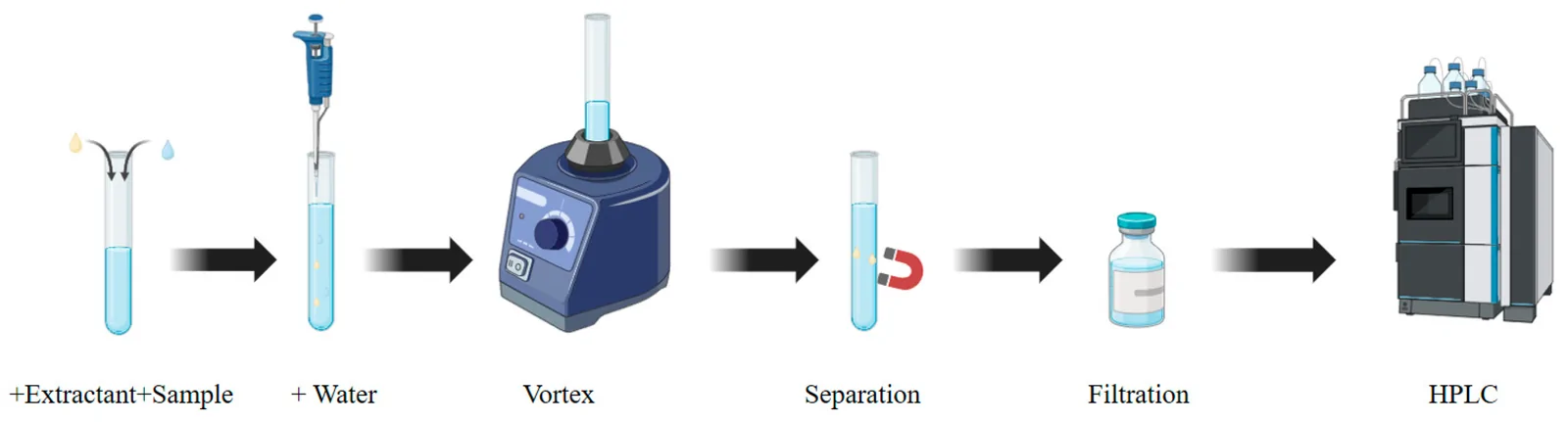
Schematic representation of the DLLME-HPLC method.

An MDES was employed as the extraction solvent (300 μL), while the sample extract served as the dispersive solvent (1300 μL). Both solutions were introduced into a 15 mL centrifuge tube, followed by the addition of 7 mL of water to induce phase separation. The mixture was vortex-mixed for 60 s to ensure adequate dispersion and efficient mass transfer. Subsequently, the MDES phase, selectively enriched with TFs, was rapidly isolated in an external magnetic field using a neodymium magnet. The collected MDES phase was quantitatively transferred to a microcentrifuge tube and diluted to a final volume of 300 μL with HPLC-grade acetonitrile prior to instrumental analysis (Figure 1).

## 3. Results and Discussion

### 3.1. Optimizing DLLME-HPLC Conditions

The extraction parameters were systematically optimized by examining the effects of fatty acid type, hydroxide type, molar ratio, MDES volume, BBS type, BBS volume, water volume, NaCl mass volume fraction, and pH. Preliminary experiments were systematically performed to first define viable condition ranges, then evaluate their interactive effects, and finally determine the optimal setup for DLLME-HPLC. A univariate optimization approach was adopted, with one parameter varied while the remaining experimental conditions were maintained constant. Unless otherwise specified, nonanoic acid was used as the fatty acid, Fe(OH)_3_ as the hydroxide, the fatty acid/Fe(OH)_3_ molar ratio was 5:1, the MDES volume was 300 μL, DMI was used as the BBS at 1500 μL, and the water volume was 7 mL. Cereal samples spiked at 0.01 μg g^−1^ were used to optimize the DLLME–HPLC conditions. Each experiment was conducted in triplicate.

#### 3.1.1. Optimization of Fatty Acid Type

**Figure 2:**
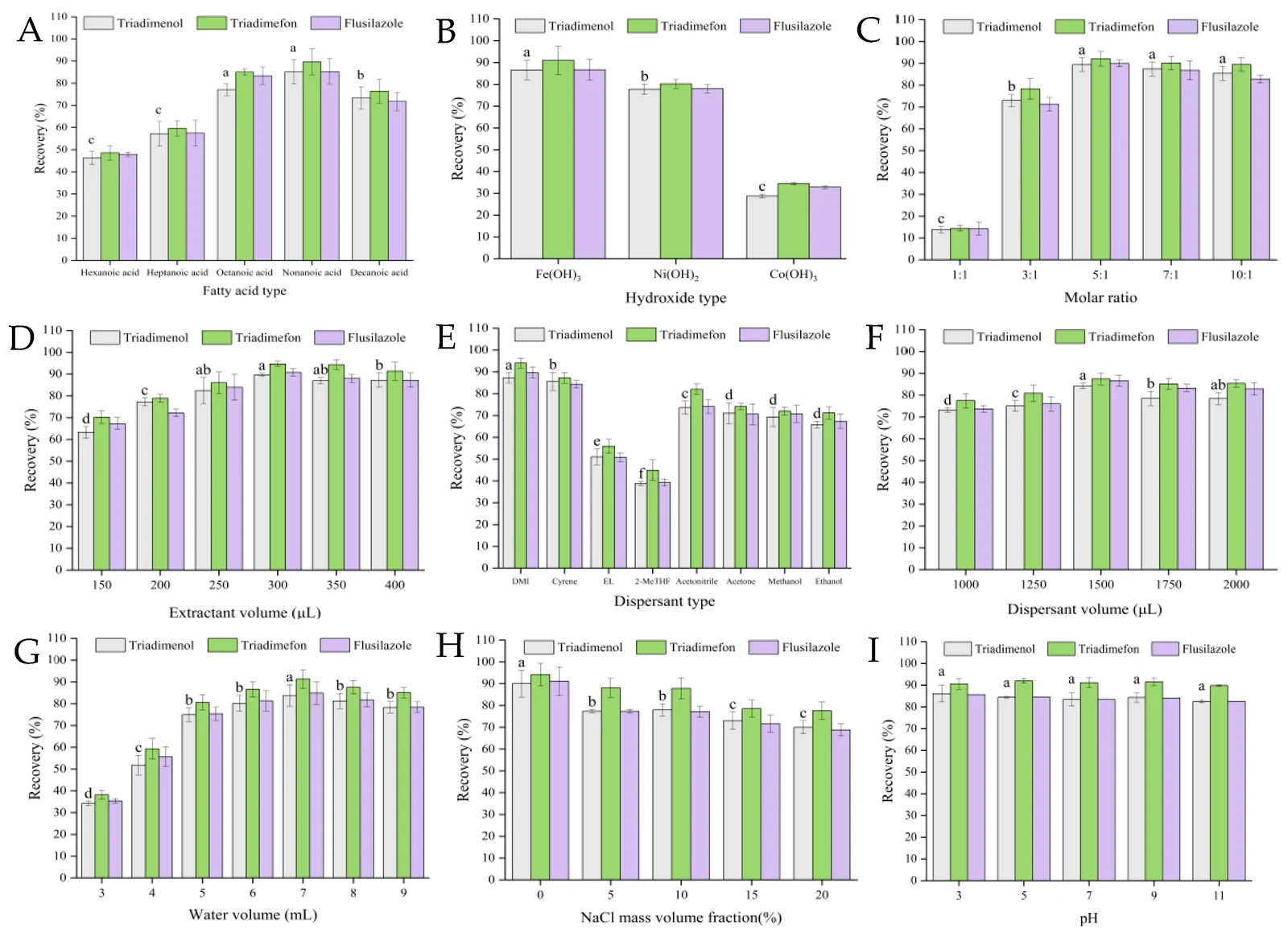
Optimization of DLLME-HPLC extraction conditions: (**A**) fatty acid type, (**B**) hydroxide type, (**C**) molar ratio, (**D**) MDES volume, (**E**) BBS type, (**F**) BBS volume, (**G**) water volume, (**H**) NaCl mass volume fraction, and (**I**) pH. Lowercase letters represent significant (*p* < 0.05).

Fatty acids in MDESs primarily bind to target pesticides [15]. To select an appropriate fatty acid for MDES preparation, experiments were conducted using pentanoic, hexanoic, heptanoic, octanoic, nonanoic, and decanoic acids. As shown in Figure 2A, the recovery gradually increased with increasing carbon chain length from pentanoic to nonanoic acid and subsequently decreased. The MDES prepared with pentanoic acid exhibited high water solubility, preventing effective collection of the extractant phase. Hexanoic, heptanoic, and octanoic acids were relatively polar, resulting in weaker interactions with TFs. In contrast, decanoic acid exhibited high viscosity, thereby increasing mass-transfer resistance and hindering the partitioning of TFs into the MDES phase. Thus, nonanoic acid was considered the most suitable fatty acid.

#### 3.1.2. Optimization of Hydroxide Type

The MDES can be rapidly separated from the aqueous phase within seconds in an external magnetic field, eliminating the need for time-consuming centrifugation. To select an optimal MDES that combines rapid, efficient extraction with green, safe characteristics, experiments were conducted with Fe(OH)_3_, Ni(OH)_2_, and Co(OH)_3_. As shown in Figure 2B, the MDES prepared with Fe(OH)_3_ achieved the highest recovery. The MDESs prepared with Ni(OH)_2_ and Co(OH)_3_ exhibited significantly weaker magnetic responsiveness than that of the MDES prepared with Fe(OH)_3_, resulting in extractant loss and, consequently, lower recoveries. In addition, Ni and Co exhibit higher toxicity, posing potential risks of contamination to both operators and analytical systems during handling, whereas Fe is an essential trace element with low physiological toxicity [16]. Therefore, Fe(OH)_3_ was considered the metal hydroxide for MDES preparation.

#### 3.1.3. Optimization of Molar Ratio

The molar ratio of components in MDESs is a critical factor in balancing extraction capability and separation efficiency [17]. To determine the optimal molar ratio for MDES preparation, experiments were conducted using nonanoic acid-to-ferric hydroxide molar ratios of 1:1, 3:1, 5:1, 7:1, and 10:1. As shown in Figure 2C, the recovery of TFs increased significantly as the molar ratio increased from 1:1 to 5:1, reached a maximum at 5:1, and subsequently decreased. With increasing proportions of nonanoic acid, the viscosity of the MDES gradually decreased, facilitating enhanced contact and interactions between TFs and the MDES. However, further increases in the proportion of nonanoic acid weakened the magnetic responsiveness of the MDES, increased the difficulty of phase separation [18], and consequently reduced recovery. Thus, a molar ratio of 5:1 was considered the optimal synthesis ratio for the MDES.

#### 3.1.4. Optimization of MDES Volume

The extractant volume is a critical factor in maximizing method sensitivity while ensuring efficient extraction [19]. To determine the optimal MDES volume, experiments were conducted using volumes of 150, 200, 250, 300, 350, and 400 μL. As shown in Figure 2D, the recoveries of TFs increased progressively with increasing MDES volume from 150 to 300 μL, reached a maximum at 300 μL, and then remained nearly constant. At lower extractant volumes, the amount of MDES was insufficient to provide an adequate extraction phase, resulting in incomplete extraction and inefficient phase separation. In contrast, excessive MDES volumes reduced extractant dispersion, promoted droplet coalescence, and decreased the effective interfacial area available for mass transfer, thereby lowering extraction efficiency [20]. Therefore, 300 μL of MDES was considered the optimal extractant volume.

#### 3.1.5. Optimization of BBS Type

The type of dispersant is a critical factor influencing the dispersion behavior of MDES droplets and interfacial mass-transfer efficiency [21]. To identify a green dispersant that could effectively disperse the MDES extractant while maintaining high recovery, four BBSs, including DMI, Cyrene, EL, and 2-MeTHF, were selected and compared with conventional dispersants (acetonitrile, acetone, methanol, and ethanol). As shown in Figure 2E, among the investigated green dispersants, DMI achieved the highest recovery and outperformed the four conventional dispersants. Therefore, DMI was considered the optimal dispersant.

#### 3.1.6. Optimization of BBS Volume

The dispersant volume affects both the dispersion efficiency of the extractant and the subsequent collection of the extraction phase [22]. To determine the optimal BBS volume, DMI volumes of 1000, 1250, 1500, 1750, and 2000 μL were investigated. As shown in Figure 2F, the recovery initially increased with increasing dispersant volume, reached a maximum at 1500 μL, and subsequently decreased. At relatively low volumes, DMI was insufficient to uniformly disperse the MDES extractant into fine droplets, resulting in limited interfacial contact between the extractant and the sample solution. However, excessive DMI caused more MDES to dissolve in the dispersant phase, leading to the retention of a portion of the pesticides in the dispersant phase and consequently reducing extraction recovery [20]. Thus, 1500 μL of DMI was considered the optimal BBS volume.

#### 3.1.7. Optimization of Water Volume

The water volume affects both extractant dispersion and phase separation efficiency. To optimize this parameter, water volumes of 3, 4, 5, 6, 7, 8, and 9 mL were investigated. As shown in Figure 2G, the recovery of TFs increased with increasing water volume, reached a maximum at 7 mL, and subsequently decreased. Insufficient water volume could not effectively separate the extractant from the sample extract, resulting in an inadequate recoverable extractant and consequently reduced recoveries. In contrast, excessive water volumes weakened the dispersion of the MDES, leading to reduced extraction efficiency. Consequently, 7 mL of water was considered the optimal volume.

#### 3.1.8. Optimization of NaCl Mass Volume Fraction

In the extraction system, the addition of NaCl to the aqueous phase may enhance the extraction efficiency of target analytes through the salting-out effect [23]. To evaluate the effect of NaCl mass fraction on the recovery of TFs in the MDES system, NaCl concentrations of 0%, 5%, 10%, 15%, and 20% were investigated. As shown in Figure 2H, the highest recovery of TFs was obtained in the absence of NaCl, and the recovery gradually decreased with increasing NaCl concentration. The addition of NaCl may interfere with the hydrogen-bonding interactions between the MDES and TFs, thereby reducing extraction recovery. Therefore, NaCl was not added to the extraction system.

#### 3.1.9. Optimization of pH

The pH of the aqueous phase may influence the existing forms of analytes in the extraction system [24]. To evaluate the effect of aqueous phase pH on the recovery of TFs, experiments were conducted at pH values of 3, 5, 7, 9, and 11. As shown in Figure 2I, the recovery of TFs remained stable across the pH range of 3–11. According to the FAO dissociation constant data, triadimenol, triadimefon, and flusilazole predominantly exist in their molecular form. MDES does not undergo substantial structural disruption because of the limited solubility. Consequently, the partitioning behavior of TFs into the MDES phase is not significantly affected by pH variations, resulting in unchanged recovery. Therefore, the pH of the aqueous phase was not further adjusted during subsequent experiments.

### 3.2. Method Validation

The established method was validated under optimized conditions to confirm its linearity, sensitivity, and suitability for simultaneously determining three TFs. Standard solutions at different concentrations were prepared and spiked into rice, wheat, corn, buckwheat, and oat samples prior to analysis, with the results summarized in Table 1. Good linearity was achieved over the concentration range of 0.001–0.1 μg g^−1^, with correlation coefficients (R^2^) ranging from 0.993 to 0.999. The limits of quantification (LOQ) and detection (LOD) were determined based on signal-to-noise ratios of 10 and 3, respectively. The LOQ and LOD were 0.001 and 0.0003 μg g^−1^, respectively. Intra-day and inter-day experiments (*n* = 3) were performed to evaluate the precision of the method. The relative standard deviations (RSDs) were 1.6–4.5% for intra-day analysis and 1.7–4.8% for inter-day analysis, demonstrating satisfactory repeatability and reproducibility. The matrix effect (ME) was evaluated by comparing the slope of the matrix standard and the solvent standard. The matrix effect ranged from 86.1% to 109.8%, which was within the acceptable range recommended by the Codex Alimentarius Commission.

### 3.3. Analysis of TFs in Real Samples

The proposed method was applied to rice, wheat, corn, buckwheat, and oat samples to evaluate its feasibility for determining TFs in real matrices. Standard solutions at three concentration levels (0.001, 0.01, and 0.1 μg g^−1^) were prepared and spiked into the samples, with each sample analyzed in triplicate. The results are summarized in Table 2. The TFs’ recoveries ranged from 75.0% to 101.9%. Overall, the proposed method demonstrated good applicability for the determination of TFs in rice, wheat, corn, buckwheat, and oat samples.

### 3.4. Green Metrics

**Figure 3:**
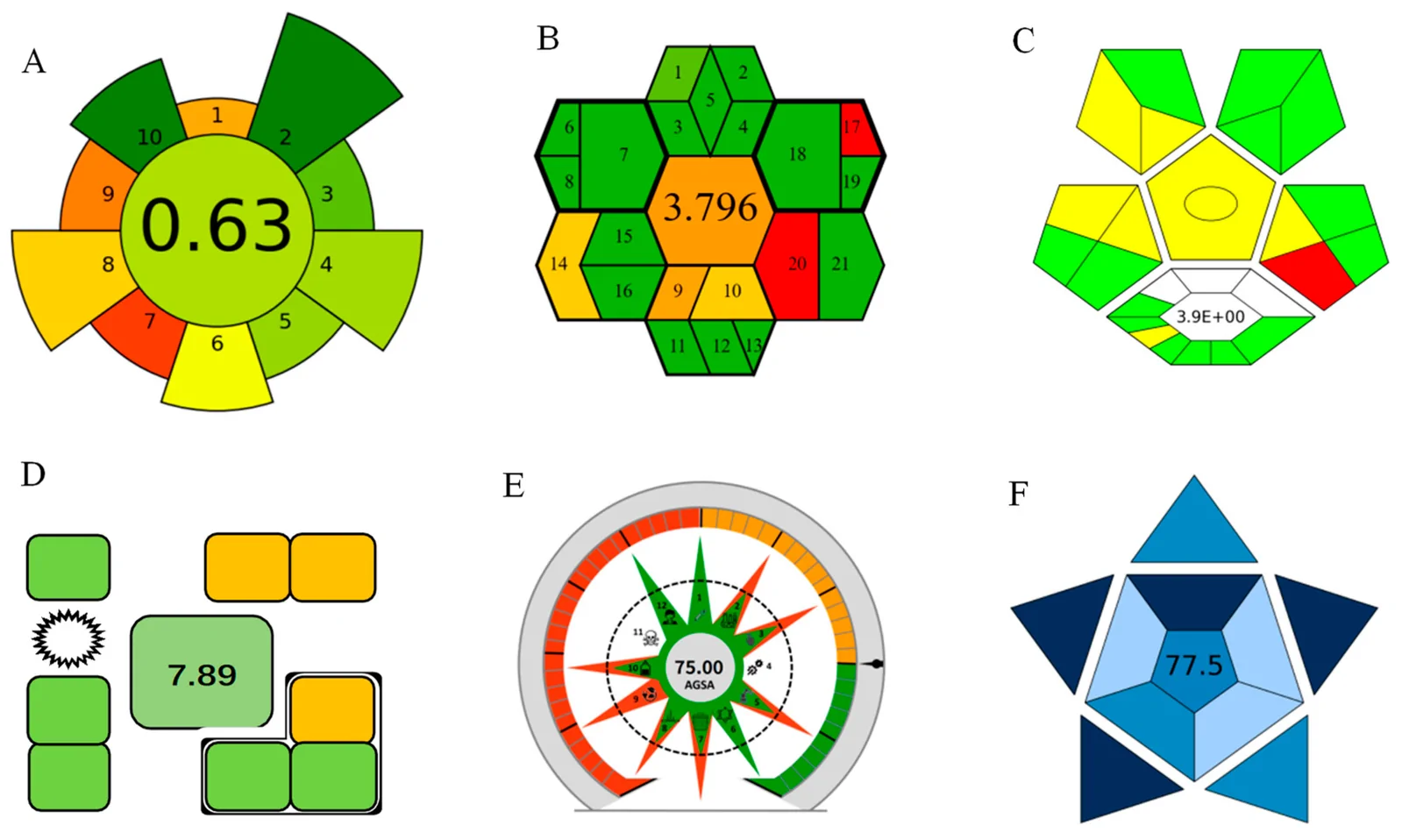
Green assessment of DLLME-HPLC: (**A**) AGREEprep, (**B**) GEMAM, (**C**) ComplexGAPI, (**D**) SPMS, (**E**) AGSA, and (**F**) BAGI.

To comprehensively assess the environmental sustainability of the developed DLLME-HPLC method, seven complementary green assessment metrics were employed. The AES evaluation [25] produced a score of 89 (Table S1), highlighting the low environmental burden of the analytical procedure. Following the ten tenets of green sample preparation, the AGREEprep assessment [26] produced a score of 0.63 (Figure 3A and Table S2), demonstrating good conformity with sustainable sample preparation practices. The GEMAM evaluation [27] produced a score of 3.796 (Figure 3B and Table S3), indicating satisfactory overall sustainability. Meanwhile, the ComplexGAPI assessment [28] generated an E-factor of 3.9 (Figure 3C and Table S4), reflecting efficient waste minimization and minimal environmental impact. The SPMS evaluation [29] produced a score of 7.89 (Figure 3D and Table S5), confirming the advantages of the method in terms of miniaturization, operational simplicity, and energy efficiency. Favorable results were also obtained with the AGSA tool [30], which produced a score of 75.0 (Figure 3E), and the BAGI tool [31], which produced a score of 77.5 (Figure 3F and Table S6). Both assessments further verified the green characteristics of the proposed analytical strategy. Collectively, these independent evaluation results consistently demonstrate that the developed DLLME-HPLC method exhibits excellent environmental sustainability and aligns well with the core principles of green analytical chemistry.

### 3.5. Comparison with Other Methods

The proposed DLLME-HPLC method was compared with representative microextraction methods reported for the determination of TFs (Table 3) [32,33,34,35]. The comparison included microextraction technique, extraction solvent, additional reagents, extraction devices, detection methods, analytical performance, and applicable sample matrices. As summarized in Table 3, most reported procedures involved relatively complex extraction systems that required toxic solvents, magnetic materials, surfactants, or additional reagents, along with auxiliary devices such as vortex mixers, centrifuges, and ultrasonic cleaners to facilitate extraction and phase separation. In comparison, the proposed method employed only MDES as the extractant and DMI as the dispersant, and the extraction process could be completed through simple vortex mixing. In terms of analytical performance, the reported methods exhibited LOQs ranging from 0.0003 to 0.009 μg mL^−1^, whereas the proposed method achieved an LOQ of 0.001 μg g^−1^, demonstrating satisfactory sensitivity. The recoveries reported for previous methods ranged from 63.4% to 119.4%, with RSDs generally below 18.5%. In comparison, the proposed method achieved recoveries of 75.0–101.9% and RSDs of 1.6–4.8%, demonstrating satisfactory accuracy and precision. Regarding applicability, previous studies have mainly focused on relatively simple liquid matrices, whereas the proposed method has been successfully applied to complex cereal matrices, including rice, wheat, corn, buckwheat, and oat. This is the first study to develop and validate an MDES-DLLME-HPLC method specifically for the determination of TFs in cereal matrices, addressing a clear gap in the current analytical methodology for these complex samples. Taken together, the developed method exhibited comparable or improved analytical performance while providing a simpler extraction procedure and broader applicability for TF determination in cereal samples.

## 4. Conclusions

Herein, a green solvent-based DLLME-HPLC method was successfully developed for the determination of TFs in cereal samples. A novel hydrophobic MDES based on nonanoic acid and ferric hydroxide was designed to serve as both the extraction medium and magnetic separation phase, achieving high extraction efficiency while eliminating the need for centrifugation. In addition, the use of BBSs as dispersive solvents effectively replaced conventional petroleum-derived solvents, further enhancing the environmental sustainability of the method. The proposed strategy exhibited satisfactory analytical performance in terms of sensitivity, precision, and applicability across different cereal matrices. Furthermore, systematic green assessment using multiple evaluation metrics demonstrated its favorable environmental profile, characterized by low solvent consumption, minimal waste generation, and simplified operation. More importantly, the combination of MDESs and BBSs provides an effective strategy for developing greener microextraction systems by simultaneously reducing hazardous solvent consumption and eliminating centrifugation during phase separation. This work not only provides a reliable analytical approach for monitoring TFs in cereals but also broadens the application of emerging green solvents in sustainable sample preparation, offering a promising strategy to advance green analytical chemistry.

## Figures and Tables

**Table 1:** Analytical performance of DLLME-HPLC.

Pesticide	Sample	Regression Equation	*R* ^2^	Intra-Day RSD (%) (*n* = 3)	Inter-Day RSD (%) (*n* = 3)	ME (%)
Triadimenol	Rice	y = 140.25x + 0.34	0.999	2.5	2.3	90.5
	Wheat	y = 140.83x + 2.63	0.999	1.8	1.7	90.8
	Corn	y = 161.61x + 2.33	0.993	1.6	4.3	104.2
	Buckwheat	y = 164.43x + 1.58	0.999	3.9	4.6	106.1
	Oat	y = 161.18x + 2.39	0.997	2.6	4.1	104.0
Triadimefon	Rice	y = 164.59x + 0.42	0.998	2.8	2.9	96.0
	Wheat	y = 147.58x + 0.33	0.996	2.0	1.9	86.1
	Corn	y = 166.37x + 0.04	0.998	4.5	4.0	97.1
	Buckwheat	y = 169.42x + 0.09	0.998	3.5	3.6	98.9
	Oat	y = 147.62x + 2.12	0.993	3.3	4.6	86.1
Flusilazole	Rice	y = 254.48x + 0.39	0.999	3.1	2.9	98.1
	Wheat	y = 239.60x + 0.39	0.997	2.4	2.4	92.4
	Corn	y = 284.62x + 1.21	0.999	3.8	4.3	109.8
	Buckwheat	y = 250.99x + 0.34	0.999	3.4	4.7	96.8
	Oat	y = 260.68x + 3.21	0.998	4.2	4.8	100.5

**Table 2:** Analysis of TFs in real samples.

TF	Spiked Level (μg g^−1^)	Rice		Wheat		Corn		Buckwheat		Oat	
		Recovery (%)	RSD (%)	Recovery (%)	RSD (%)	Recovery (%)	RSD (%)	Recovery (%)	RSD (%)	Recovery (%)	RSD (%)
Triadimenol	0.1	89.1	3.1	83.3	4.2	84.6	2.9	93.4	3.9	86.0	0.3
	0.01	93.9	1.4	77.1	4.5	92.0	2.3	94.5	2.1	84.1	2.0
	0.001	89.2	1.0	75.0	3.8	77.6	3.4	79.0	2.8	79.6	3.8
Triadimefon	0.1	91.5	3.5	88.5	4.0	86.9	3.2	95.8	4.0	91.4	3.3
	0.01	100.6	1.5	79.9	4.5	94.7	4.3	101.9	3.9	90.1	2.4
	0.001	94.0	1.4	83.9	3.6	81.1	5.7	80.0	3.8	88.1	3.6
Flusilazole	0.1	88.1	2.1	84.2	2.9	83.6	3.0	92.6	3.8	84.7	1.2
	0.01	93.0	1.2	76.9	4.8	92.4	2.6	94.2	3.1	84.9	2.8
	0.001	87.5	0.9	78.4	2.4	78.7	2.3	75.8	2.4	83.4	2.8

**Table 3:** Comparing DLLME with reported microextraction methods for determining TFs.

Microextraction Method	Extraction Solvent	Additional Reagent	Extraction Device	Detection Method	LOQ	Recovery (%)	RSD (%)	Sample Type	Ref.
LPME	SDES	H_2_SO_4_ Na_2_CO_3_	Centrifuge	HPLC-UV	0.005 μg mL^−1^	72.6–95.4%	4.7–18.5%	Water Beverage	[32]
LPME	Undecanol HFIP	-	Vortex mixer Centrifuge	HPLC-UV	0.005 μg mL^−1^	63.4–112.1%	<13.7%	Water Beverage	[33]
MSPE	Fe_3_O_4_@SiO_2_@F/NiO	Ethanol Urea	Ultrasonic cleaner Vortex mixer	HPLC-UV	0.0003 μg mL^−1^	77.9–119.4%	0.9–8.7%	Water Milk Tea	[34]
μ-SPE	CHF Sodium dodecyl sulfate	Methanol	Vortex mixer Centrifuge	HPLC-UV	0.009 μg mL^−1^	67.0–105.0%	0.6–4.3%	Water Milk Juice Beverage	[35]
DLLME	MDES	DMI	Vortex mixer	HPLC-DAD	0.001 μg g^−1^	75.0–101.9%	1.6–4.8%	Rice Wheat Corn Buckwheat Oat	This study

## Data Availability

The original contributions presented in the study are included in the article and Supplementary Materials; further inquiries can be directed to the corresponding authors.

## Supplementary Materials

The following supporting information can be downloaded at https://www.mdpi.com/article/10.3390/foods15173002/s1, Figure S1. The representative chromatogram of triadimenol, triadimefon, and flusilazole; Figure S2. Vibrating sample magnetometer analysis of MDES; Table S1. AES assessment of DLLME-HPLC; Table S2. AGREEprep assessment of DLLME; Table S3. GEMAM assessment of DLLME-HPLC; Table S4. ComplexGAPI assessment of DLLME-HPLC; Table S5. SPMS assessment of DLLME; Table S6. BAGI assessment of DLLME-HPLC.
