# Supplementary material for: Green Solvent-Based Dispersive Liquid–Liquid Microextraction Method Coupled with High-Performance Liquid Chromatography for the Determination of Triazole Fungicides in Cereal Samples
Source: Foods. 2026 Aug 26;15(17):3002. doi: 10.3390/foods15173002 (PMC13564566; doi:10.3390/foods15173002)
Supplement: Supplementary file 1 [file foods-15-03002-s001.zip › foods-4484034-supplementary.pdf]

Supplementary material

Green Solvent-Based Dispersive Liquid–Liquid Microextraction Method Coupled with High-Performance Liquid Chromatography for the Determination of Triazole Fungicides in Cereal Samples

Min Li [1], Yulin Wang [2], Huajuan Yin [2], Xu Jing [2,*] and Yunlong Li [1,*]

1 Shanxi Institute for Functional Food, Shanxi Agricultural University, Taiyuan 030031, China; li407267281@163.com

2 Houji Laboratory in Shanxi Province, College of Food Science and Engineering, Shanxi Agricultural University, Taiyuan 030031, China; 15161869838@163.com (Y.W.); 16635424019@163.com (H.Y.)

* Correspondence: x.jing@vip.163.com (X.J.); liyunlong125@126.com (Y.L.);

Tel.: +86-03546288325 (X.J.); +86-03517122507 (Y.L.)

Number of pages: 9

Number of tables: 6

Number of figures: 2

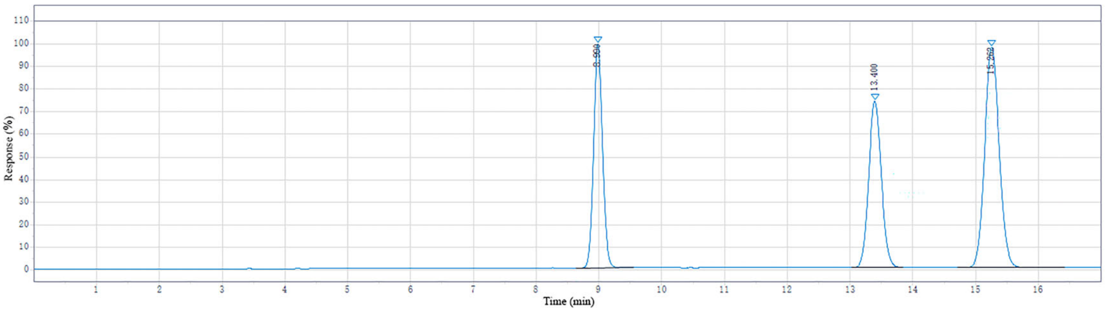

Figure S1. The representative chromatogram of triadimenol, triadimefon, and flusilazole.

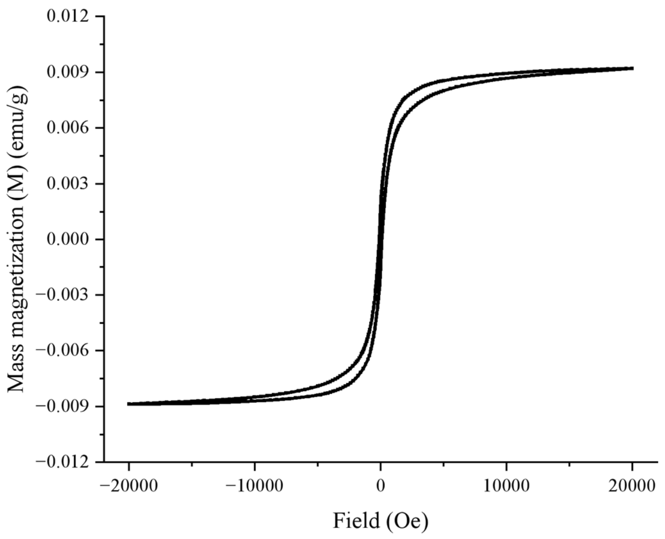

Figure S2. Vibrating sample magnetometer analysis of MDES.

This relatively low viscosity indicates that MDES has favorable fluidity and mass transfer characteristics, making it a low-viscosity solvent well suited for DLLME applications.

Table S1. AES assessment of DLLME-HPLC.

Penalty points			
1. Reagent			
1.1. MDES	Amount	< 10 mL	1
	Hazard type	Less severe hazard	1
	Hazard amount	1	1
			Total PPs = 2
2. Instruments	HPLC-DAD		
2.1. Energy		< 0.1 kWh per sample	0
2.2. Occupational hazard		Gas or vapor	3
			Total PPs = 3
3. Waste			
	3.1. Waste amount	1–10 mL (g)	3
	3.2. Waste treatment	No treatment	3
			Total PPs = 6
Total penalty points			11
Eco-scale score			100–11 = 89

Table S2. AGREEprep assessment of DLLME.

	Criterion	Evaluation information	Score	Weight
1	Sample preparation placement	*On-site*	0.75	2
2	Hazardous materials Sustainability	0.005 g or mL	0.87	5
3	Renewability and reusability of materials	> 75 % of reagents and materials are sustainable or renewable	0.87	2
4	Waste	0.885 g or mL	0.75	4
5	Size economy of the sample	Mass or volume of the sample: 1 g or mL	1.00	2
6	Sample throughput	9 samples h^{-1}	0.55	3
7	Integration and automation	Sample prep. steps: 4 step; Manual systems	0.37	2
8	Energy consumption	100 W per sample	0.49	4
9	Post-sample preparation configuration for analysis	Liquid chromatography	0.19	2
10	Operator’s safety	No hazards or no exposure	1.00	3

Table S3. GEMAM assessment of DLLME-HPLC.

Sections	Evaluation content	Assessment results	Score	Weight
Sample information	The position of the analytical device	On-line	1.5	0.3
	Whether the sample is destroyed during sample collection	No	2	
	The range of sample collection	Microextraction	1.5	
	The size of the sample	8.5 mL	2.0	
	Storage of the sample	Under room temperature	2.0	
	The amounts of sample		9.0	
Reagents information	Description of the ideal green derivatization	No derivatization	1.0	0.2
	The amounts of reagents	0.35 mL	1.0	
	The score of the reagents		2.0	
Method information	Analysis parameters	3	0.8	0.15
	Sample throughput (per h)	9	1.5	
	Number of main steps in the analysis process	3 or fewer	2.0	
	Ratio of the mass of sustainable and renewable materials to the total mass of materials used	Materials are all sustainable, renewable and are used multiple times.	2.0	
	Economic benefits of the method	0.5	1.0	
	The score of the method		7.3	
Instrument information	Most energy-intensive	HPLC-DAD	2.5	0.1
	Energy consumed per sample analyzed	1.5 kWh		
	Automation of instruments	Automatic	3.0	
	Miniaturization of instruments	Miniaturized	3.0	
	The score of the instruments		8.5	
Waste information	Waste treatment	No treatment	0	0.2
	The amounts of wastes	0.885 g	1.0	
	The score of wastes		1.0	
Operator	Hermetic sealing of the analytical process	Emission	0	0.05
	Noise generating of analytical process	60.0 dB	5.0	
	The score of operators		5.0	
Total score			3.796	

Table S4. ComplexGAPI assessment of DLLME-HPLC.

Category		Evaluation
Sample preparation	Collection (1)	Online
	Preservation (2)	None
	Transport (3)	None
	Storage (4)	Under normal conditions
	Type of method: direct or indirect (5)	Simple procedures
	Scale of extraction (6)	Micro-extraction
	Solvents/reagents used (7)	Green solvents/reagents used
	Additional treatments (8)	None
Reagents and solvents	Amount (9)	1–10 mL (1–10 g)
	Health hazard (10)	Slightly toxic and irritating.
	Safety hazard (11)	No special hazards
Instrumentation	Energy (12)	< = 1.5 kWh per sample
	Occupational hazard (13)	Hermetic sealing of the analytical process
	Waste (14)	< 1 mL (< 1 g)
	Waste treatment (15)	No treatment
	Quantification (16)	Yes
Yield and conditions	Yield (I)	Not applicable
	Temperature/time (II)	Not applicable
Relation to Green Economy	Number of rules met (III)	5–6
Reagents and solvents	Health hazard (IVa)	Slight toxic, slight irritant, NFRA = 0/1
	Safety hazard (IVb)	NFPA = 0/1, No special hazards
Instrumentation	Technical setup (Va)	Common setup
	Energy (Vb)	< = 1.5 kWh per sample
	Occupational hazard (Vc)	Hermetic sealing of the analytical process
Workup and purification	End products workup, purification (VIa)	None or simple process
	Purity (VIb)	Not applicable
E-factor	E-factor input (VII)	3.9

Table S5. SPMS assessment of DLLME.

Category	Metric parameter	Value of the parameter	Assigned numeric score	Color score & qualitative rating
Sample information	Sample amount	≤ 10 mL	5	Green (successful)
Extractant information	Amount of extractant	0.35 mL	12	Orange (acceptable)
	Nature of extractant	MDES (Alternative degradable)	12	Orange (acceptable)
Procedure information	Number of steps	2 ≤ x ≤ 4 step Addition of extractant and emulsifier to the sample	5	Orange (acceptable)
	Extraction time	5 ≤ x ≤ 15 step	5	Orange (acceptable)
	Additional steps after extraction	No additional steps	10	Green (successful)
Energy consumption	Dispersion/stir	Vortex	3	Orange (acceptable)
	Separation	No centrifuge	5	Green (successful)
	Temperature	Room temperature	5	Green (successful)
Waste	Total waste	0.725 g	10	Green (successful)
Reusability mark		Non-reusable extractant	Yes	
Sample throughput		Multiple sample/mark	Yes	
Final score			69 (out of 95) 7.89 (out of 10)	

Table S6. BAGI assessment of DLLME-HPLC.

	Principle	Subparts	Score
1	Type of analysis	Quantitative and confirmatory	10
2	Number of analytes that are simultaneously determined	Multi-element analysis for 2–5 compounds of the same chemical class	5
3	Analytical technique-instrumentation	Simple instrumentation is available in most labs	7.5
4	Number of samples that can be simultaneously treated	2–12	5
5	The sample preparation scale	Miniaturized extraction sample preparation (SPME, DLLME, MEPS, SBSE, d-SPE, FPSE, etc.)	5
6	Number of samples that can be analyzed per hour	5–10	7.5
7	Type of reagents and materials	Common commercially available reagents	10
8	Requirement for preconcentration	No preconcentration was required. The required sensitivity and/or legislative criteria are met directly.	10
9	The automation degree	Semi-automated with common devices	7.5
10	The amount of the sample	< 100 µL bioanalytical samples, < 10 mL food/environmental	10
